# Variation in youth and young adult homicide rates and their association with city characteristics in Latin America: the SALURBAL study

**DOI:** 10.1016/j.lana.2023.100476

**Published:** 2023-03-20

**Authors:** Amélia Augusta de Lima Friche, Uriel Moreira Silva, Usama Bilal, Olga L. Sarmiento, Maria Angélica de Salles Dias, Francisco Javier Prado-Galbarro, Roberto Briceño-León, Marcio Alazraqui, Ana V. Diez-Roux, Waleska Teixeira Caiaffa

**Affiliations:** a Observatory for Urban Health in Belo Horizonte, School of Medicine, Federal University of Minas Gerais, Belo Horizonte, Minas Gerais, Brazil; b Department of Speech, Language and Hearing Sciences, School of Medicine, Federal University of Minas Gerais, Belo Horizonte, Minas Gerais, Brazil; c Urban Health Collaborative, Dornsife School of Public Health, Drexel University, Philadelphia, PA, USA; d Department of Epidemiology and Biostatistics, Dornsife School of Public Health, Drexel University, Philadelphia, Pennsylvania, USA; e School of Medicine, Universidad de los Andes, Bogotá, Colombia; f Population Health Research Center, National Institute of Public Health, Cuernavaca, Mexico; g Department of Research, Hospital Infantil de México Federico Gómez, Mexico City, Mexico; h Federal University of Ceará, Fortaleza, Brazil & Venezuelan Observatory of Violence, Caracas, Venezuela; i Instituto de Salud Colectiva, Universidad Nacional de Lanús, Buenos Aires, Argentina

**Keywords:** Homicide, Urban health, Latin America, Mortality, Urban form, Social factors

## Abstract

**Background:**

Latin America and the Caribbean (LAC) is one of the most urbanized and violent regions worldwide. Homicides in youth (15–24 years old, yo) and young adults (25-39yo) are an especially pressing public health problem. Yet there is little research on how city characteristics relate to homicide rates in youth and young adults. We aimed to describe homicide rates among youth and young adults, as well as their association with socioeconomic and built environment factors across 315 cities in eight LAC countries.

**Methods:**

This is an ecological study. We estimated homicide rates in youth and young adults for the period 2010–2016. We investigated associations of homicide rates with sub-city education and GDP, Gini, density, landscape isolation, population and population growth using sex-stratified negative binomial models with city and sub-city level random intercepts, and country-level fixed effects.

**Findings:**

The mean sub-city homicide rate per 100,000 in persons aged 15–24 was 76.9 (SD = 95.9) in male and 6.7 (SD = 8.5) in female, and in persons aged 25–39 was 69.4 (SD = 68.9) in male and 6.0 (SD = 6.7) in female. Rates were higher in Brazil, Colombia, Mexico and El Salvador than in Argentina, Chile, Panama and Peru. There was significant variation in rates across cities and sub-cities, even after accounting for the country. In fully adjusted models, higher sub-city education scores and higher city GDP were associated with a lower homicide rate among male and female (rate ratios (RR) per SD higher value in male and female, respectively, 0.87 (CI 0.84–0.90) and 0.90 (CI 0.86–0.93) for education and 0.87 (CI 0.81–0.92) and 0.92 (CI 0.87–0.97) for GDP). A higher city Gini index was associated with higher homicide rates (RR 1.28 (CI 1.10–1.48) and 1.21 (CI 1.07–1.36) in male and female, respectively). Greater isolation da was also associated with higher homicide rates (RR 1.13 (CI 1.07–1.21) and 1.07 (CI 1.02–1.12) in male and female, respectively).

**Interpretation:**

City and sub-city factors are associated with homicide rates. Improvements to education, social conditions and inequality and physical integration of cities may contribute to the reduction of homicides in the region.

**Funding:**

The 10.13039/100010269Wellcome Trust [205177/Z/16/Z].

**Research in context**

**Evidence before this study**

Although the estimated number of homicide victims is decreasing globally, homicide rates remain unacceptably high in many countries, including in Latin America and the Caribbean (LAC), characterized by a high level of urbanization and inequality. Yet few studies have examined how city-level factors are related to urban homicides. Identifying and understanding these factors and their relationships with homicide in youth and young people is essential to support urban policies to reduce homicides in the region.

**Added value of this study**

Our study uses unique harmonized data to characterize homicide rates among youth and young adults in about 85% of all cities of 100,000 inhabitants in eight LAC countries. We found very high rates overall: the mean sub-city homicide rate per 100,000 in persons aged 15–24 was 76.9 in male and 6.7 in female, and in persons aged 25–39 was 69.4 in male and 6.0 (SD = 6.7) in female. We also observed important heterogeneity not only between countries but also across cities within a country and across sub-city areas within a city. Cities with a better social and built environment such as higher education levels, higher GDP, lower income inequality, and lower landscape isolation had lower homicide rates in youth and young adults.

**Implications of all the available evidence**

The high levels and the large variability of homicide rates across cities in youth and young adults in cities of the region highlight the importance of homicides as a public health and social problem. The associations we observed also suggest that urban policies related to education, income inequality, and urban planning may play an important role in reducing the very high homicide rates in cities LAC.

## Introduction

Violence is a major public health and social problem across the globe. Worldwide, there are more than 1.3 million deaths annually as a result of violence including self-directed, interpersonal and collective violence, corresponding to about 2.5% of total global mortality.^1^ In the year 2000, worldwide, homicides were responsible for more casualties than those occurring in armed conflicts (8.8 and 5.2 per 100,000 individuals, respectively).^2^ Globally, the estimated number of homicide victims in 2012 was approximately 475,000 (6.7/100,000 inhabitants),^1^ decreasing to 405,000 in 2017.^1^ However, the decrease has not been equally distributed and has been less pronounced in low and middle-income countries, some of which have experienced stable or even increasing homicide rates.^1^ Violence is also spatially patterned, exhibiting variation between countries, between regions within countries, and between cities.^1^ Accelerated urbanization has been linked to growing violence.^3^ In low- and middle-income countries, the historical, social and economic process of urbanization has occurred in ways that foster inequities across and within cities with important implications for the development,^4^ and for levels of violence in cities.

The impact of violence varies by age and gender. Violence is the fourth leading cause of death in youth and young adults (15–44 years old) worldwide. Homicide, or interpersonal violence, accounts for about a third of all violent deaths for men in this age group.^1^ Men are especially affected by homicides: they account for 82% of all homicide victims.^1^ Among women, homicides are the most extreme form of violence along a continuum of gender-based discrimination and abuse.^5^ Thus, homicides in women are a distinct phenomenon, and in many ways, different from homicides in men. However due to lower rates, homicides in women are often invisible to society.^1^

Latin America and the Caribbean (LAC), is one of the most urbanized regions in the world, with 80% of its population living in cities, and is also among the most violent: eight out of the 10 most violent countries in the world are in LAC.^6^ Although LAC accounts for only 8% of the world's population, 37% of all the world's homicides occurred in the region in 2012. The region accounted for more than a third of all homicides (36%) worldwide in the year 2012, and had the highest homicide rate (28.5/100,000) of all regions.^1^ The region also has experienced a fast and disorganized urbanization process,^4^ leading to pronounced urban inequalities. Political and economic crises, the consolidation of organized crime, internal conflicts, and the marked inequities exacerbated by economic policies have also contributed to very high levels of urban homicides in the region.^4^^,^^7^

Despite the high levels of homicides observed in Latin America and in particular in cities of the region, few studies have examined how various city-level factors are related to levels of urban violence particularly among youth and young adults, the group with the higher rates among all ages.^1^ Although some evidence suggests that a number of features of urban places including city size and density,^8^ connectivity,^4^ poverty, inequities and unemployment,^9^ availability of weapons,^4^ presence of gangs and drug trade^5^^,^^10^ and alcohol abuse^4^ may be related to youth homicide rates, few studies have investigated how features of urban places relate to youth homicides across diverse Latin American cities. Identifying and understanding these factors and their relationships with homicide in youth and young people is important to support urban policies to mitigate homicides in Latin America.^11^

We used harmonized data from over 300 cities in the region to: (1) describe levels and variability in homicide rates among youth and young adults across all cities with 100,000 residents or more in eight countries of Latin America; and (2) investigate how city and sub-city social, economic and environmental characteristics are related to homicide rates.

## Methods

This study is part of the SALURBAL^11^ (*Salud Urbana en America Latina)* project. The study identified all cities of 1000,000 residents or more in 2010 in participating countries. Cities were defined as agglomerations of country-specific administrative units (*municipios, comunas, distritos, corregimientos, or partido*s) that encompassed the urban extent, identified using satellite images.^11^^,^^12^ The country-specific administrative units that compose cities are henceforth referred to as sub-cities. We included a total of 1205 sub-cities nested within 315 cities in eight countries, representing around 85% of all cities with 100,000 or more residents in these countries in 2010. The other cities (15%) had incomplete data for some exposures and were excluded.

We obtained data on homicide deaths from the vital registration systems of each country. Homicides were identified using the Global Health Estimates (GHE) codes 1580 (violence) and 1600 (other intentional injuries), corresponding to International Classification of Diseases version 10 (ICD10) codes X85-Y09, Y871 and Y35 (Supplementary Table S2).^13^ Injuries of ill-defined intent (e.g. Y10–Y34 and Y872) were redistributed to specific types of injury using established methods from the literature described elsewhere,^13^ and the ones identified as homicides by these methods were also considered in our study. To address the lack of complete coverage of all deaths, we computed an undercounting correction factor using an ensemble of death distribution methods described elsewhere^8^ and used the correction factor in descriptive and regression analyses. The deaths of individuals of unspecified age were not included in the study.

We focused on youth (ages: 15–24) and young adult (ages: 25–39) homicide deaths. We aggregated homicide counts at the sub-city level from 2010 to 2015 for Argentina, Brazil, Chile, Colombia, Costa Rica, Mexico and Peru; from 2012 to 2016 for Panama and from 2010 to 2014 for El Salvador. As denominators, we used postcensal population projections or intercensal population estimates obtained from national census bureaus or equivalent.^8^

We investigated features of city social and built environments (Supplementary Table S1). Measures were defined at the city or sub-city level depending on the construct of interest. We selected features that have been hypothesized to be related to homicides in prior works.^6^^,^^7^ Measures of the social environment (SOE) included sub-city population educational attainment, city Gross Domestic Product (GDP) and city income-based Gini index. Population educational attainment was defined at the sub-city level to capture spatial heterogeneity within cities. The education score^14^ was defined as the sum of the Z-scores of 2 census-based indicators: % of population with more than 25 years of age with at least secondary education and % of population with more than 25 years of age with at least university education. Higher values of the score represent better education within cities. City GDP was derived from estimates of gridded GDP per capita for 2010, expressed in 2011 purchasing power parity (PPP) US dollars.^15^ City Gini index data was obtained from national surveys,^16^ with higher values representing more inequality.

Built environment (BE) indicators included measures of four dimensions: city population density (to capture heterogeneity with cities) and city isolation of urban development, city population size, and city population growth. Population density was measured by dividing the total population by the built up area in 2010.^17^ Isolation measures the relative spatial isolation of development relative to other development in the area. To assess isolation, we used the area-weighted mean nearest neighbor distance across patches for 2012 defined as the mean distance (in meters) to the nearest urban patch within the geographic boundary weighted by the area of each patch, with higher values reflecting higher levels of isolation.^18^ Total population was defined as the mean of the city population counts over the aggregation years for each country. Population growth was computed as the relative difference in the city population between the first aggregation year and five years afterwards.

We computed the undercounting-corrected homicide rates per 100,000 inhabitants for each sub-city, sex (female or male assigned in death certificate), age category and year. We also computed aggregated homicide rates per 100,000 inhabitants pooling across all years. We then examined the distributions of sub-city rates over time, by country and sex.

To assess variability in homicide rates within cities, between cities within countries and between countries, we fitted a sex-stratified multilevel linear model with the logarithm of homicide rates (pooling across ages) as the outcome and with random intercepts at the city and country level. The variability across levels was then evaluated by calculating the percent of variance at each level.

We used sex-stratified negative binomial models to estimate the association of city and sub-city social and built environment characteristics with homicide rates. We modelled the count of deaths in each age group and each sub-city unit summed across all years as a function of city and sub-city characteristics with a random intercept for each city and sub-city. Models included dummy variables representing the young adult age group and country-level fixed effects. The logarithm of the population multiplied by the undercounting correction factor, divided by 100,000 and summed across all years was used as an offset.

We fit a series of models: the first included each exposure separately, then two models including all SOE and BE exposures in the same model, respectively, and a final model including all exposures together. All exposures were standardized to have a mean of 0 and a standard deviation of 1, with city size also being log-transformed prior to standardization. All analyses were conducted with the package glmmTMB in the R software environment.

### Ethics approval statement

The SALURBAL study protocol was approved by the *Drexel University Institutional Review Board* with ID #1612005035 and by appropriate site-specific IRBs.

### Role of the funding source

The funding sources had no role in the analysis, writing or decision to submit the manuscript.

## Results

Table 1 shows the distributions of homicide rates and social and built environment characteristics by country. The mean sub-city homicide rate per 100,000 was 76.9 (SD = 95.9) in males and 6.7 (SD = 8.5) in females aged 15–24, and in persons aged 25–39 was 69.4 (SD = 68.9) in males and 6.0 (SD = 6.7) in females. Mean homicide rates per 100,000 inhabitants for males were 10–11 times larger than the rates for females in the same age range. When aggregating across the full sample, youth (15–24 years old) homicide rates were higher than young adult (25–39 years old) homicide rates in both sexes, although the differences across the age groups were small and not consistent across countries (Table 1). There was also significant heterogeneity across countries: El Salvador had the highest mean homicide rates, followed by Colombia and Brazil; whereas Argentina, Chile and Peru had the lowest rates (only one Peruvian city, Lima, was included in the sample). Mexico had intermediate rates.

**Table 1:** Summary statistics of homicide rates (pooled across all years) and social and built environment characteristics in cities, by country.

Variables	Total	AR	BR	CL	CO	MX	PA	PE^a^	SV
**Sample characteristics**									
# of cities	315	26	152	21	17	92	3	1	3
# of sub-cities	1205	101	422	81	40	406	82	51	22
**Aggregated homicide rates per 100,000 hab.**									
Youth, females (15–24 yo)	7.59	1.94	9.16	1.31	11.38	7.92	6.95	0.76	23.25
Young adults, females (25–39 yo)	7.00	1.88	7.80	1.53	10.46	8.22	4.40	0.70	15.04
Youth, males (15–24 yo)	101.00	23.56	158.38	14.61	160.05	59.97	93.61	5.18	171.30
Young adults, males (25–39 yo)	84.46	18.02	103.06	14.85	150.68	85.68	69.77	5.92	170.64
**Sub-city homicide rates per 100,000 hab.: means (standard deviations)**									
Youth, females (15–24 yo)	6.71 (8.54)	1.70 (1.51)	8.59 (7.09)	1.09 (1.66)	10.91 (6.69)	6.78 (9.32)	6.29 (12.60)	0.37 (0.76)	21.65 (10.07)
Young adults, females (25–39 yo)	6.02 (6.71)	1.67 (1.18)	8.11 (5.80)	1.88 (2.60)	9.57 (5.35)	5.78 (6.29)	4.45 (11.24)	0.43 (0.69)	17.74 (8.53)
Youth, males (15–24 yo)	76.99 (95.09)	18.77 (13.26)	135.69 (115.36)	14.60 (11.13)	130.71 (96.50)	40.69 (48.27)	79.42 (81.25)	3.66 (6.82)	181.01 (80.79)
Young adults, females (25–39 yo)	69.44 (69.89)	14.55 (9.95)	98.20 (67.16)	15.49 (10.53)	130.09 (73.71)	61.43 (66.97)	63.96 (60.74)	4.53 (7.73)	176.76 (68.86)
**Social environment characteristics: means (standard deviations)**									
Sub-city education (index)	−0.46 (1.56)	−0.29 (1.60)	−0.56 (1.06)	−0.66 (1.34)	−0.47 (0.83)	−0.94 (1.38)	0.76 (2.08)	2.52 (1.87)	−1.15 (1.69)
City GDP (x10^3^ 2011 PPP USD)	15.58 (11.34)	18.01 (10.63)	15.12 (8.24)	20.67 (16.16)	8.83 (4.71)	15.59 (14.60)	22.11 (11.54)	31.26 (0.00)	7.85 (1.70)
City Gini (index)	0.49 (0.08)	0.40 (0.03)	0.55 (0.05)	0.41 (0.03)	0.46 (0.03)	0.45 (0.06)	0.49 (0.03)	0.40 (0.00)	0.41 (0.02)
**Built environment characteristics: means (standard deviations)**									
City Isolation (m)	95.61 (40.81)	88.00 (23.59)	89.74 (33.04)	95.65 (36.32)	111.53 (74.63)	106.47 (47.12)	73.02 (6.00)	63.25 (0.00)	69.19 (4.35)
City Population density (x10^2^ hab./km^2^)	69.23 (34.55)	55.23 (11.59)	63.56 (24.36)	70.93 (17.60)	167.76 (45.00)	61.45 (22.48)	66.51 (12.35)	158.43 (0.00)	119.80 (19.72)
City size (x10^5^ hab.)	4.13 (10.65)	5.22 (14.59)	3.75 (10.13)	2.94 (6.66)	4.22 (4.75)	4.22 (11.22)	3.90 (4.84)	47.73 (0.00)	3.93 (4.62)
City Population growth (% over 5 years)	6.54 (3.52)	6.14 (2.49)	5.96 (3.81)	6.59 (2.41)	5.88 (3.87)	7.62 (3.27)	7.91 (2.79)	8.12 (0.00)	8.06 (2.33)

AR = Argentina, BR = Brazil, CL = Chile, CO = Colombia, MX = Mexico, PA = Panama, PE = Peru, SV = El Salvador; yo = years old; hab = inhabitants; PPP= Purchasing Power Parity; m = meters.

a The standard deviations of city-level Peru variables is 0 due to the sample only having one city (Lima) for this country.

**Fig. 1:**
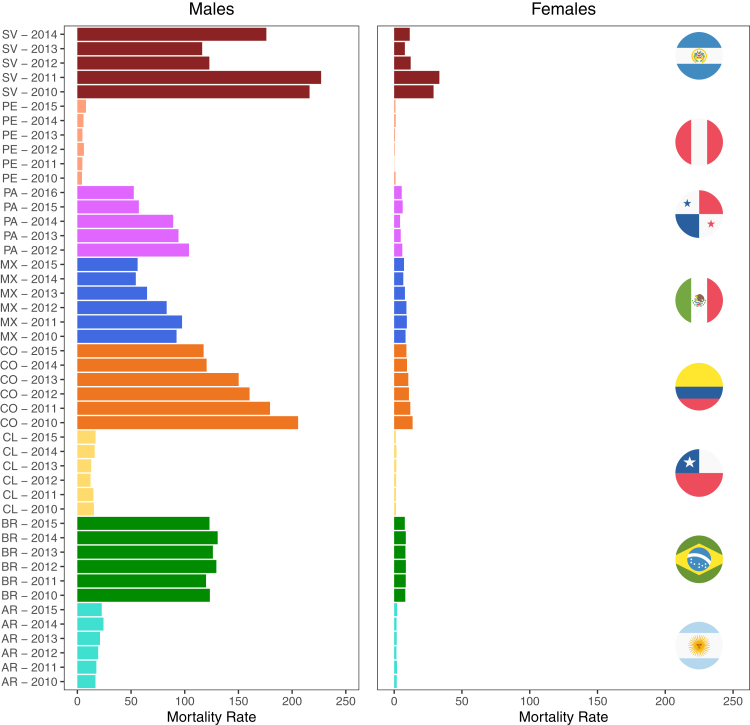
**Homicide rates per 100,000 inhabitants aggregated over age ranges and all sub-city units for each country and year combination, stratified by sex.** AR = Argentina, BR = Brazil, CL = Chile, CO = Colombia, MX = Mexico, PA = Panama, PE = Peru, SV = El Salvador.

Fig. 1 shows homicide rates by country and year in males and females for both age groups combined. Homicide rates decreased over time (between 2010 and 2015) in Colombia, Mexico and El Salvador but were approximately stable in the other countries. In analyses pooling across years, there was evidence of substantial heterogeneity in homicide rates: of the total variance in log homicide rates across sub-city units in males, 24% was between sub-cities within cities, 20% was between cities within countries and 56% was between countries. Analogously, for females, 41% of the total variance was between sub-cities, 11% was between cities and 48% was between countries (Supplementary Fig. S1).

Table 2 shows associations of city factors with homicide rates stratified by sex. Correlations between SOE and BE factors were moderate to low: the strongest correlations were observed for city size and density (0.5) and city size and isolation (−0.5) (Supplementary Fig. S2). In males in single exposure models, higher sub-city education, higher city GDP and higher city population growth were associated with lower homicide rates, whereas higher city income inequality, higher city population density, higher city isolation, and larger city size were associated with higher homicide rates. These associations were similar in females, except that population density was not associated with homicide rates in females and associations of larger city size with higher rates and larger growth with lower rates were weaker than in males and not statistically significant.

**Table 2:** Estimated rate ratios (95% confidence intervals) of sub-city homicide rates associated with a 1SD higher value of social and built environment characteristics in males and females in single exposure and multiple exposure models.

	Single exposure	All social environment	All built environment	Fully adjusted
**Males**				
***Social environment***				
Education	0.87 (0.84–0.90)	0.87 (0.84–0.90)		0.87 (0.84–0.90)
GDP	0.83 (0.78–0.89)	0.86 (0.80–0.92)		0.87 (0.81–0.92)
Gini	1.43 (1.25–1.64)	1.40 (1.22–1.60)		1.28 (1.10–1.48)
***Built environment***				
Isolation	1.11 (1.05–1.18)		1.16 (1.09–1.24)	1.13 (1.07–1.21)
Population density	1.22 (1.07–1.39)		1.12 (0.98–1.27)	1.06 (0.93–1.20)
City size	1.19 (1.04–1.35)		1.29 (1.12–1.48)	1.16 (0.99–1.35)
Population growth	0.91 (0.85–0.98)		0.94 (0.88–1.01)	0.97 (0.90–1.03)
**Females**				
***Social environment***				
Education	0.89 (0.86–0.93)	0.89 (0.86–0.93)		0.90 (0.86–0.93)
GDP	0.90 (0.85–0.95)	0.92 (0.87–0.97)		0.92 (0.87–0.97)
Gini	1.23 (1.11–1.37)	1.22 (1.10–1.36)		1.21 (1.07–1.36)
***Built environment***				
Isolation	1.07 (1.02–1.12)		1.09 (1.04–1.15)	1.07 (1.02–1.12)
Population density	1.01 (0.92–1.12)		0.96 (0.87–1.07)	0.93 (0.84–1.03)
City size	1.06 (0.96–1.16)		1.14 (1.02–1.27)	1.05 (0.93–1.18)
Population growth	0.96 (0.91–1.01)		0.96 (0.91–1.01)	0.98 (0.93–1.03)

The outcome is homicide rates at the sub-city-level, education is at the sub-city-level and all the other exposures are at the city-level. All models assume a Negative Binomial distribution with constant dispersion parameter and include sub-city-level and city-level random intercepts, country-level fixed effects and a dummy for age. A logarithmic link was used, and an offset equal to the log of the corresponding population divided by the undercounting correction factor (itself multiplied by 100,000) was included. All exposures were standardized to have a mean of 0 and a standard deviation of 1.

The association of social environment variables, education, GDP and Gini, with homicide rates did not change when all three social environment variables were adjusted for each other in males or in females (Table 2). In males, when all built environment variables were adjusted for each other, associations of population density with higher rates and city growth with lower rates were weakened and no longer statistically significant, whereas greater isolation and larger city size remained strongly associated with higher homicide rates. Similar results were observed in females: when all built environment variables were adjusted for, greater isolation and larger city size remained associated with higher rates although associations were weaker than in males.

In the fully adjusted model (Table 2), we found that homicide rates among both males and female remained significantly associated with social environment variables with associations being similar in males and females. Specifically, higher sub-city education scores and higher city GDP were associated with a lower homicide rate among males and females (rate ratios per SD higher education score (RR) 0.87 (CI 0.84–0.90) and 0.90 (CI 0.86–0.93) in males and females, respectively; and rate ratios per SD higher GDP 0.87 (CI 0.81–0.92) and a 0.92 (CI 0.87–0.97) in males and females, respectively). A higher city Gini index was associated with higher homicide rates (RR per 1SD higher Gini 1.28 (CI 1.10–1.48) and 1.21 (CI 1.07–1.36) in males and females, respectively). Of the built environment variables, only isolation of development remained significantly associated with higher homicide rates after full adjustment (RR per 1SD higher isolation value 1.13 (CI 1.07–1.21) and a 1.07 (CI 1.02–1.12) in males and females, respectively). The association of higher city size with higher homicide rates was weakened after adjustment for social environment variables, although the point estimate still reflected a substantial association in males (1.16 higher rates for each 1SD higher population size) but a weaker association in females (1.05 higher rates for each 1SD higher population size). Population density was not significantly associated with homicide rates in the fully adjusted models, although point estimates suggested a weakly positive association in males and a weakly inverse association in females.

## Discussion

We studied the distribution of homicide deaths among persons 15–39 years across 1205 sub-cities in 315 cities of 100,000 persons or more in eight countries of Latin America. This represents 85% of all cities with 100,000 residents or more in these countries, as of 2010. As expected, the homicide death rate was substantially higher in males than in females but there was important heterogeneity across cities and across sub-city units within cities. For both sexes, higher levels of sub-city education and city GDP were associated with lower homicide rates, while higher city income inequality and higher city landscape isolation were associated with higher homicide rates.

We found a high overall homicide rate for residents aged 15–39 years in the eight LAC countries with little difference between youth and young adults. These rates were much higher than the European average and the global rates for 2012^1^ (3.8/100.000 inhabitants). This is not surprising given the high homicide rates previously reported in Latin American countries,^6^ especially in urban areas.^1^^,^^4^ It clearly illustrates the high burden of homicide in youths and young adults in cities of the region. Other work has shown that rates among persons aged 15–44 years comprise around 60% of all homicide deaths in Latin America.^1^ The high impact of homicides on young people is a consistent pattern across all national income levels, but consistently with our findings is especially marked in low- and upper middle-income countries.^1^ Lack of economic and employment opportunities, drug trafficking, abusive alcohol consumption, lack of leisure activities, and interactions with law enforcement are some of the potential determinants of homicides in young people.^1^^,^^2^^,^^4^^,^^19^ Deaths among youth have large economic costs and a great impact on families and society.^20^

We also found that homicide rates among males were about 11 times higher than in females in the same age group. However, it is important to highlight that our data is based on biological sex, which may not fully reflect the nuances of gender-based violence that permeate homicides on those who self-identify as women. The intersection of a lack of economic opportunities, interactions with law enforcement, and a culture of toxic masculinity, which imposes on men the need to demonstrate their force and power, may contribute to higher homicide rates among men.^6^ In countries like Colombia, Mexico, and El Salvador, internal conflicts also contribute to homicides, especially in young males.^19^ In the case of homicides among males, in general, the victim and the perpetrator are both males, and most of the time, they do not have a previous relationship.^1^^,^^3^^,^^19^^,^^21^ In women, homicide often represents the final outcome of a history of repeated abuse and silent violence, occurring since infancy and inside households. This is especially marked in LAC, where there still is a deeply rooted patriarchal culture leading to *machismo*. As a result, most homicides among women are femicides, that is, the perpetrators are often their male partner or relatives and the crimes occur inside homes.^4^^,^^5^^,^^21^ Violence against women also generates a climate of fear and restricts economic and other opportunities for women.^3^ Although the magnitude of the differences between the sexes reinforces the importance of homicides in youth and young male, the impact of homicides in females deserves equal attention.^5^ Gender-stratified data could contribute to the study of gender inequality and, to do so, gender-sensitive data is dearly needed to consider the gender social implications in homicide rates.

Although the age and sex patterns were approximately consistent across all countries, we also observed significant heterogeneity in levels of homicide not only between countries but also across cities and sub-city units within countries. Moreover, we found that city and sub-city characteristics were significantly associated with levels of homicide, often similarly, in males and females. The association between better socioeconomic conditions and homicide rates is consistent with other work showing that better living conditions are linked to lower homicide rates.^1^^,^^2^^,^^4^^,^^22^ Levels of sub-city education may reflect educational and job opportunities for youth and young adults as well as life expectations. The absence of opportunities may favor the engagement of young people and young adults in illegal activities such as drug trafficking, and encourage violent behavior.^1^^,^^4^^,^^6^^,^^21^

We also found that a higher city GDP was associated with lower mortality rates, even after adjusting for country factors. This is consistent with other studies showing a negative association between GDP and homicide mortality in LAC, including Colombia^23^ and Brazil^24^**.** We add to this work by demonstrating that higher city GDP is associated with lower homicide rates even after adjusting for education, inequality and other city and country factors. The mechanisms that could link higher GDP to lower homicide rates include access to job opportunities and better living conditions. We also found that larger city income inequality was associated with a higher homicide rate. This is consistent with previous observations showing that homicides tend to occur more frequently in areas in which both wealthy and extremely poor individuals can be found.^4^^,^^9^ Many other studies have found associations of homicides with worse social conditions and inequalities,^1^^,^^3^^,^^5^^,^^19^^,^^21^^,^^22^^,^^25^^,^^26^ although few have examined differences across cities.^8^^,^^26^

After adjustment for all other covariates, we found that higher isolation of development was associated with higher homicide rates among both males and females. Isolated areas may be socially and economically disadvantaged,^27^^,^^28^ which could undermine life opportunities because residents are isolated from neighborhoods with greater resources. A study of Chicago's neighborhood commuting network suggested that neighborhood isolation and disconnectedness from other neighborhoods is linked to more violence.^27^ There is also evidence showing that poor connectivity, topographic difficulties, poor access to social services, insufficient police protection and precariousness of the state of law existing in isolated areas may be related to the involvement of young adults and even children with drugs and firearms, as well as the presence of alternative mechanisms of social control such as gangs,^4^^,^^9^ all of which may impact homicide rates.

Although other associations of built-environment factors with homicide rates were not statistically significant, point estimates did suggest that larger cities have higher homicide rates independent of other characteristics in both males and females consistent with previous studies.^1^^,^^4^^,^^8^^,^^20^ Previous studies have shown that fast city growth, especially in cities without adequate planning and government support, can result in a disorganized environment and consequently, increased violence.^6^^,^^20^^,^^28^ Moreover, uncontrolled city growth, i.e. urbanization without planning, is often linked to residential segregation, which can aggravate inequities.^26^^,^^29^ However, we did not find an association of faster city growth with homicide rates; in fact, in single exposure models, faster city growth was associated with lower homicide rates, which may have reflected confounding by city social and economic environmental factors. Furthermore, we did not find any association between homicide rates and population density. In contrast, other studies in Latin America have found both lower^30^ and higher homicide rates associated with higher population density.^29^

An important strength of our study is that it encompassed nearly all cities of 100,000 residents or more in eight LAC countries, representing a wide variety of social and economic conditions and leveraged unique harmonized data on homicides as well as social and built environment data from a large multicentric study.^12^ We applied state-of-the-art demographic methods to correct for incomplete coverage, as reported in previous studies,^8^ and redistributed ill-defined injuries using the methods of the GHE study. Although undercounting correction and redistribution might be insufficient in places where data is sparse, we have reason to suspect that this might not be the case in our study, since we limit ourselves to areas with at least 100,000 inhabitants.^8^ Our analysis was ecological, so we could not account for individual-level factors such as education, income or employment. Also, we only had information about sex which did not allow us to address directly the gender-social implications of homicides. We also acknowledge that there is a time gap between the year when exposures were collected (2010) to the year that outcomes were collected (2010–2016). We explored a range of possible determinants and did not attempt to identify causal chains or draw firm causal inferences as this is precluded by the study design and nature of the data. Nevertheless, our results are compatible with causal processes that merit further exploration.

We found high rates of homicides in cities of Latin American countries, with especially high rates being observed in males and in cities of El Salvador, Brazil, Mexico, Colombia and Panama. Even after accounting for country, we found wide variations in homicide rates across cities. In general, cities with higher education levels, higher GDP, lower income inequality and lower landscape isolation had lower homicide rates. Our findings point towards the possible role of urban policies related to education, income inequality, and urban planning in reducing the very high homicide rates in cities of LAC.

## Contributors

ADR, WTC, OLS, MA, and AALF obtained funding for the study. AALF, WTC, UMS and ADR conceived the study. UMS conducted the statistical analyses with support from AALF, WTC, ADR and UB. AALF and UMS wrote the first version of the manuscript, WTC and ADR revised it. All authors helped in the interpretation of results, reviewed the manuscript providing critical inputs, and approved the manuscript. Members of the SALURBAL Group contributed to the overall conduct of the study as well as to data collection, data processing and data harmonization for this paper.

## Data sharing statement

The SALURBAL project welcomes queries from anyone interested in learning more about its dataset and potential access to data. To learn more about SALURBAL's dataset, visit the SALURBAL project website or contact the project at salurbal@drexel.edu.

## Declaration of interests

None of the authors had any conflict of interest.

## Appendix A Supplementary data

Supplementary material

Abstract_ESP_disclaimer ok

Abstract_PORT_disclaimer ok
