# Supplementary material for: Variation in youth and young adult homicide rates and their association with city characteristics in Latin America: the SALURBAL study
Source: Lancet Reg Health Am. 2023 Mar 20;20:100476. doi: 10.1016/j.lana.2023.100476 (PMC10033737; doi:10.1016/j.lana.2023.100476)
Supplement: Supplementary material [file mmc1.pdf]

Supplementary Material to MS34: Variation in youth and young adult homicide rates and their association with city characteristics in Latin America: the SALURBAL study

Negative Binomial GLMM specification

The “fully adjusted” model in the paper corresponds to

$$Y_{ij} | X_{ij} \sim \text{Negative Binomial}(\mu_{ij}, \phi),$$

where Y_{ij} is the homicide count of young adults and youth at the ith sub-city and jth city, $i = 1, \dots, I$, $j = 1, \dots, J$ (there are a total of I = 1,205 subcities and J = 315 cities in the sample). Here, X_{ij} represents all the conditional information in the model (i.e. exposures, adjustments and random effects) and $\phi \geq 0$ is a dispersion parameter such that the smaller its value, the smaller the overdispersion is (the limiting case $\phi \to +\infty$ corresponds to the Poisson, i.e. equidisperse, case). Since a logarithm link was assumed, the log of the conditional mean μ_{ij} satisfies

$$\log(\mu_{ij}) = O_{ij} + \beta_0 + \alpha_i + \alpha_j + \beta_1 A_{ij} + \sum_{k=1}^{7} \gamma_k C_{ij}^{k} + \sum_{l=1}^{3} \theta_l S_j^{l} + \sum_{p=1}^{4} \eta_p B_j^{p},$$

where $O_{ij} = \log(N_{ij}/(U_{ij} \cdot 10^5))$ is the model offset (U_{ij} is the undercounting correction factor and N_{ij} is the population of young adults and youth in the ith sub-city and jth city), β_0 is the model intercept, α_i is a random interceptat the ith sub-city, α_j is a random intercept at the jth city, A_{ij} is an indicator of age range (0 = youth, 1 = young adults) with coefficient β_1, C_{ij}^{k} are country-level indicators (reference = Peru) with coefficients γ_k, S_j^{l} are the social environment exposures (educational achievement score, GDP and Gini index) at the jth city with coefficients θ_l and B_j^{p} are the built environment exposures (isolation of urban patches, aggregation, city size and population growth) at the jth city with coefficients η_p. Note that the models are stratified by sex, so there is both a “male” and a “female” version of this model.

As stated in the paper, the above definition adopted for the offset O_{ij} and the use of a logarithmic link allows for modelling the undercounting corrected homicide rates $R_{ij} = Y_{ij} \cdot U_{ij} 10^5 / N_{ij}$ indirectly as a function of the model outcome Y_{ij} and the offset O_{ij} as defined above, since

$$\log(R_{ij}) = \log\left(Y_{ij} \cdot \frac{U_{ij} 10^5}{N_{ij}}\right) = \log(Y_{ij}) + \log\left(\frac{U_{ij} 10^5}{N_{ij}}\right) = \log(Y_{ij}) - O_{ij} \Leftrightarrow \log(Y_{ij}) = \log(R_{ij}) + O_{ij}.$$

Finally, the parameterization used here for the Negative Binomial is

$$P(Y_{ij} = k) = \frac{\Gamma(k + \phi)}{\Gamma(k + 1)\Gamma(\phi)} \left(\frac{\phi}{\phi + \mu_{ij}}\right)^{\phi} \left(\frac{\mu_{ij}}{\phi + \mu_{ij}}\right)^{k}, \qquad k = 0, 1, \dots$$

where $\Gamma(x) \coloneqq \int_0^{+\infty} t^{x-1} e^{-t} dt$ denotes the Gamma function. This parameterization ensures that the conditional expectation of Y_{ij} given X_{ij} is $E(Y_{ij} | X_{ij}) = \mu_{ij}$ and that the corresponding conditional variance is $V(Y_{ij} | X_{ij}) = \mu_{ij} + \mu_{ij}^2 / \phi$.

Log-rates clustering analyses model specification

Our model for the logarithm of homicide rates is

$$\log(R_{ijk}) = \beta_0 + \alpha_j + \alpha_k + \varepsilon_{ijk}, \qquad \varepsilon_{ijk} \sim iid\ N(0, \sigma_I^2),$$

where $R_{ijk} = 10^5 \cdot Y_{ijk} \cdot U_{ij} / N_{ijk}$ is the homicide rate at the ith sub-city, jth city and kth country, Y_{ijk} are the corresponding homicide counts, U_{ijk} is the undercounting correction factor and N_{ijk} is the population at risk (note that here we are stratifying for sex and have aggregated undercounting-corrected counts and population across both age ranges – young adults and youth). We also assume that $\alpha_j \sim N(0, \sigma_J^2)$, $\alpha_k \sim N(0, \sigma_K^2)$ and that α_j, α_k and ε_{ijk} are all independent of each other for all i, j and k.

Here, we define the total model variance as

$$\sigma_T^2 := \sigma_I^2 + \sigma_J^2 + \sigma_K^2$$

and therefore the percentage of the variance attributed to subcities, cities and cities are, respectively,

$$\sigma_{subcity}^2 = \frac{\sigma_I^2}{\sigma_T^2} \times 100\%, \qquad \sigma_{city}^2 = \frac{\sigma_J^2}{\sigma_T^2} \times 100\%, \qquad \sigma_{country}^2 = \frac{\sigma_K^2}{\sigma_T^2} \times 100\%.$$

Additional methods and results

Table S1 contains the definition, source and geographic unit of each variable used in the paper, Table S2 contains the correspondence between ICD-10 and GHE 2015 codes used for defining (and redistributing) homicide deaths in the main text, Table S3 contains summary statistics (median + IQRs) for all of the variables used in the paper, Figure S1 contains boxplots of the logarithm of homicide rates aggregated over the sample years by country and gender and Figure S2 contains the correlation matrix between all the exposures used in the main (multilevel negative binomial regression) analyses.

Table S1. Overall structure of the data used in this paper. Each variable was collected and harmonized by SALURBAL's team based on data from the corresponding source.

Variable	Type	Availability	Data Source
Homicide counts for young adults and youth	outcome	sub-city	Vital registration systems
% of population aged > 25 w/ at least secondary education	SE exposure (component of education score)	sub-city	National censuses
% of population aged > 25 w/ at least universitary education	SE exposure (component of education score)	sub-city	National censuses
Gross Domestic Product	SE exposure	city	Gennaioli et al. (2013)
Income-based Gini index	SE exposure	city	National household surveys
Area-weighted mean nearest-neighbor distance	BE exposure (proxy for isolation)	city	Global Urban Footprint Project
Population density	BE exposure (proxy for aggregation)	city	Global Urban Footprint Project
Total population	BE exposure (proxy for city size)	city	Population projections
Population growth	BE exposure (proxy for city growth)	city	Population projections
Age (0 = 25-39 yrs old) (1 = 15-24 yrs old)	covariate	sub-city	Created by the authors
Sex (0 = female) (1 = male)	covariate	sub-city	Created by the authors
Projected population for young adults and youth	covariate	sub-city	Population projections
Undercounting correction factor	covariate	city	Vital registration systems and population projections

Table S2. Correspondence between the codes in the Global Health Estimates (GHE) 2015 version and 4-digit ICD-10 codes within the Tier 2 GHE 2015 category “B. Intentional Injuries”.

GHE code	ICD-10 codes	GHE category name
1570	X60-X849, Y870	Self-inflicted injuries
1580	X85-Y099, Y871	Violence
1590	Y36-Y369	War
1600	Y35-Y359	Other intentional injuries

Source: Input dataset: Global Health Estimates 2016: Deaths by Cause, Age, Sex, by Country and by Region, 2000-2016. Geneva, World Health Organization. https://www.who.int/healthinfo/global_burden_disease/GHE2016_Input-Data.7z

Note: the corresponding code for the Tier 2 GHE 2015 category “B. Intentional Injuries” is 1560.

Table S3. Summary statistics of homicide rates (pooled across all years) and social and built environment characteristics in cities, by country.

VARIABLES	TOTAL	AR	BR	CL	CO	MX	PA	PE*	SV
**Sample characteristics**									
# of cities	315	26	152	21	17	92	3	1	3
# of sub-cities	1,205	101	422	81	40	406	82	51	22
**Aggregated homicide rates per 100,000 hab.**									
Youth, women (15-24 yo)	7·59	1·94	9·16	1·31	11·38	7·92	6·95	0·76	23·25
Young adults, women (25-39 yo)	7·00	1·88	7·80	1·53	10·46	8·22	4·40	0·70	15·04
Youth, men (15-24 yo)	101·00	23·56	158·38	14·61	160·05	59·97	93·61	5·18	171·30
Young adults, men (25-39 yo)	84·46	18·02	103·06	14·85	150·68	85·68	69·77	5·92	170·64
**Subcity homicide rates per 100,000 hab.: medians (interquartile ranges)**									
Youth, women (15-24 yo)	4·14<br>(9·49)	1·48<br>(2·25)	7·20<br>(8·45)	0·00<br>(1·86)	8·74<br>(7·86)	4·26<br>(8·85)	0·00<br>(10·49)	0·00<br>(0·50)	24·90<br>(14·31)
Young adults, women (25-39 yo)	4·50<br>(7·81)	1·65<br>(1·33)	7·20<br>(7·26)	1·32<br>(2·86)	8·39<br>(6·85)	4·72<br>(8·04)	0·00<br>(6·50)	0·00<br>(0·81)	17·73<br>(9·73)
Youth, men (15-24 yo)	39·62<br>(83·04)	16·11<br>(13·32)	96·53<br>(151·30)	11·46<br>(12·52)	96·88<br>(97·11)	26·82<br>(35·68)	57·94<br>(87·68)	0·00<br>(3·71)	172·69<br>(83·31)
Young adults, men (25-39 yo)	47·82<br>(78·47)	13·35<br>(12·65)	80·62<br>(92·12)	13·50<br>(12·46)	106·88<br>(90·36)	43·60<br>(48·63)	54·26<br>(64·01)	2·31<br>(4·59)	171·07<br>(68·35)
**Social environment characteristics: medians (interquartile ranges)**									
Subcity education (index)	-0·71<br>(1·72)	-0·59<br>(1·52)	-0·61<br>(1·41)	-1·01<br>(1·20)	-0·59<br>(1·26)	-1·19<br>(1·82)	0·18<br>(1·88)	1·77<br>(2·33)	-1·70<br>(1·46)
City GDP (x10^3 2011 PPP USD)	14·73<br>(10·55)	17·47<br>(10·19)	19·19<br>(12·34)	15·47<br>(11·85)	7·80<br>(4·21)	13·00<br>(5·72)	24·36<br>(11·37)	31·26<br>(0·00)	6·99<br>(1·52)
City Gini (index)	0·50<br>(0·12)	0·40<br>(0·05)	0·55<br>(0·05)	0·40<br>(0·04)	0·46<br>(0·03)	0·45<br>(0·05)	0·50<br>(0·03)	0·40<br>(0·00)	0·41<br>(0·02)
**Built environment characteristics: medians (interquartile ranges)**									
City Isolation (m)	82·43<br>(30·55)	77·86<br>(20·45)	80·27<br>(23·05)	81·91<br>(28·14)	85·60<br>(41·94)	91·74<br>(41·78)	71·76<br>(5·89)	63·25<br>(0·00)	71·32<br>(3·93)
City Population density (x10^2 hab./km^2)	58·98<br>(27·05)	51·24<br>(16·37)	55·33<br>(26·61)	64·14<br>(23·96)	164·39<br>(52·45)	58·74<br>(18·98)	73·33<br>(10·85)	158·43<br>(0·00)	127·28<br>(18·63)
City size (x10^5 hab.)	1·51<br>(2·24)	1·73<br>(1·98)	1·19<br>(1·81)	1·10<br>(0·96)	2·31<br>(2·69)	1·84<br>(3·04)	1·14<br>(4·20)	47·73<br>(0·00)	1·30<br>(4·02)
City Population growth (% over 5 years)	6·12<br>(4·02)	5·92<br>(2·13)	5·49<br>(3·66)	5·86<br>(2·32)	4·75<br>(5·09)	7·49<br>(3·28)	8·55<br>(2·74)	8·12<br>(0·00)	7·77<br>(2·31)

Footnote: *The interquartile ranges of city-level Peru variables is 0 due to the sample only having one city (Lima) for this country.

Legend: AR = Argentina, BR = Brazil, CL = Chile, CO = Colombia, MX = Mexico, PA = Panama, PE = Peru, SV = El Salvador, yo = years old, hab = inhabitants, PPP = Purchasing Power Parity, m = meters.

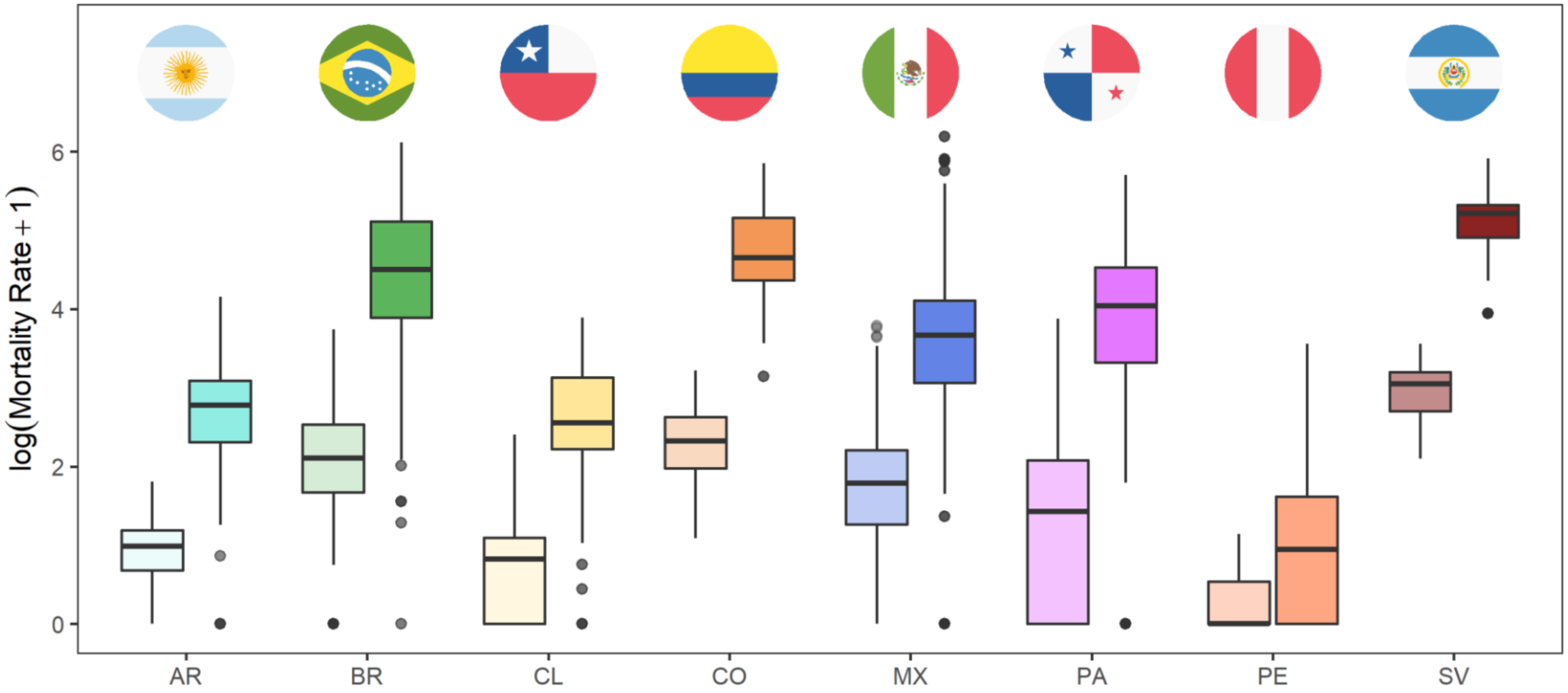

Figure S1. Boxplots of the logarithm of homicide rates* aggregated over the sample years by country and sex.

*Prior to log-transforming, we added 1 to the homicide rates so that the log-rate equals 0 if the aggregated rates are also 0.

Legend: Solid horizontal lines are the medians, lower and upper hinges are the 1st and 3rd quartiles, lower and upper vertical lines are at least 1.5 times the interquartile range in length and lower and upper dots are outliers and inliers. AR = Argentina, BR = Brazil, CL = Chile, CO = Colombia, MX = Mexico, PA = Panama, PE = Peru, SV = El Salvador. For each country, estimates for women are on the left (softer shades) and estimates for men are on the right (darker shades).

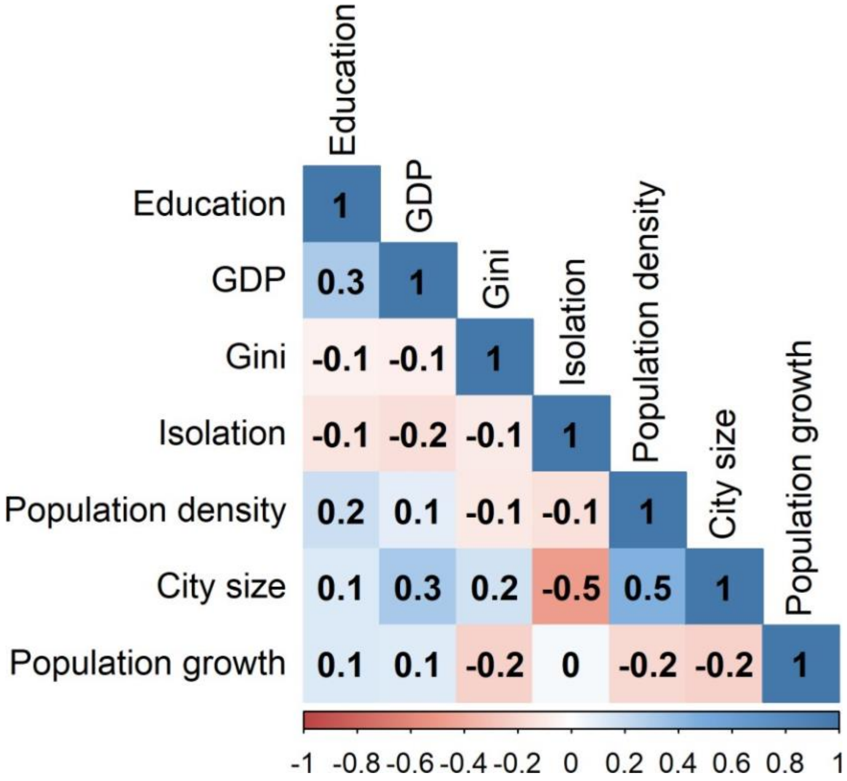

Figure S2. Correlation matrix between all of the exposures used in the main text.
