# Supplementary material for: Variation in youth and young adult homicide rates and their association with city characteristics in Latin America: the SALURBAL study
Source: Lancet Reg Health Am. 2023 Mar 20;20:100476. doi: 10.1016/j.lana.2023.100476 (PMC10033737; doi:10.1016/j.lana.2023.100476)
Supplement: Abstract_ESP_disclaimer ok [file mmc2.docx]

***Editorial Disclaimer:*** *This translation in Portuguese was submitted by the authors and we reproduce it as supplied. It has not been peer-reviewed. Our editorial processes have only been applied to the original abstract in English, which should serve as a reference for this manuscript.*

**RESUMEN**

**Contexto:** Latinoamérica y el Caribe (LAC) es una de las regiones más urbanizadas y más violentas del mundo. Homicidios en jóvenes (15-24 años) y adultos jóvenes (25-39 años) son un problema de salud pública de gran urgencia. Sin embargo hay poca investigación sobre como las características de la ciudad se relacionan con tasas de homicidio entre jóvenes y adultos jóvenes. Nuestro objetivo fue describir las tasas de homicidio entre jóvenes y adultos jóvenes, bien como su asociación con factores del ambiente socioeconómico y construido a lo largo de 315 ciudades en ocho países de LAC.

**Métodos:** Este es un estudio ecológico. Fueron estimadas tasas de homicidio de jóvenes y adultos jóvenes durante el periodo de 2010-2016. Fueron investigadas asociaciones de tasas de homicidio con educación y PIB, Gini, densidad, aislamiento de las manchas urbanas, población y crecimiento poblacional a nível de subciudad utilizando modelos binominales negativos estratificados por sexo con interceptaciones aleatorias a nível de subciudad y ciudad, y efectos fijos a nivel de país.

**Resultados:** La tasa media de homicidio a nivel de subciudad por 100,000 en personas entre 15-24 años fue de 76.9 (SD=95.9) en hombres y 6.7 (SD=8.5) en mujeres, y en personas entre 25-39 años fue 69.4 (SD=68.9) en hombres y 6.0 (SD= 6.7) en mujeres. Las tasas fueron más altas en Brasil, Colombia, México y El Salvador que en Argentina, Chile, Panamá y Peru. Hubo variación significativa en tasas entre ciudades y subciudades, mismo después de considerado el país. En modelos totalmente ajustados, scores más altos para educación a nível de subciudad y PBI más alto a nivel de ciudad fueron asociados a tasa de homicidio más baja entre hombres y mujeres (proporciones de tasa (RR) por valor más alto de desviación estándar en hombres y mujeres, respectivamente, 0.87 (CI 0.84-0.90) y 0.90 (CI 0.86-0.93) para educación y 0.87 (CI 0.81-0.92) y 0.92 (CI 0.87-0.97) para el PBI). Un indice de Gini a nível de ciudad más alto fue asociado a tasas de homicidio más altas (RR 1.28 (CI 1.10-1.48) y 1.21 (CI 1.07-1.36) en hombres y mujeres, respectivamente). Mayor aislamiento de desarrollo fue asociado a tasas de homicidio más altas (RR 1.13 (CI 1.07-1.21) and 1.07 (CI 1.02-1.12) en hombres y mujeres, respectivamente).

**Interpretación:** Factores a nível de ciudad y subciudad fueron asociados a tasas de homicidio. Mejoras en la educación, condiciones sociales e inequidad y integración física de ciudades pueden contribuir para la reducción de los homicidios em la región.

**Financiamiento:** Wellcome Trust [205177/Z/16/Z]

**Palabras clave:** Homicidio; Salud Urbana; Latinoamérica, Mortalidad; Forma urbana; Factores sociales
