# Supplementary material for: Variation in youth and young adult homicide rates and their association with city characteristics in Latin America: the SALURBAL study
Source: Lancet Reg Health Am. 2023 Mar 20;20:100476. doi: 10.1016/j.lana.2023.100476 (PMC10033737; doi:10.1016/j.lana.2023.100476)
Supplement: Abstract_PORT_disclaimer ok [file mmc3.docx]

***Editorial Disclaimer:*** *This translation in Portuguese was submitted by the authors and we reproduce it as supplied. It has not been peer-reviewed. Our editorial processes have only been applied to the original abstract in English, which should serve as a reference for this manuscript.*

**Resumo**

**Contexto:** A região da América Latina e do Caribe (LAC) é uma das mais urbanizadas e violentas do mundo. Homicídios em jovens (15-24 anos) e em adultos jovens (25-39 anos) são um problema de saúde pública premente. Entretanto, há poucas pesquisas sobre como as características das cidades se relacionam com as taxas de homicídio nessas faixas etárias. Nosso objetivo foi descrever as taxas de homicídio entre jovens e adultos jovens, bem como sua associação com fatores socioeconômicos e do ambiente construído em 315 cidades em oito países da ALC.

**Métodos:** Este é um estudo ecológico. Estimamos as taxas de homicídios em jovens e adultos jovens para o período 2010-2016. Investigamos associações de taxas de homicídio com educação e PIB de subcidades, índice de Gini, densidade populacional, isolamento, tamanho da população e crescimento populacional usando modelos binomiais negativos estratificados por sexo com interceptações aleatórias em nível de cidade e subcidade e efeitos fixos em nível de país.

**Resultados:** A taxa média de homicídios nas subcidades por 100.000 habitantes em pessoas de 15 a 24 anos foi de 76,9 (DP = 95,9) em homens e de 6,7 (DP = 8,5) em mulheres, e em pessoas de 25 a 39 anos foi de 69,4 (DP = 68,9) em homens e 6,0 (DP= 6,7) no sexo feminino. As taxas foram mais altas no Brasil, Colômbia, México e El Salvador comparadas as taxas de Argentina, Chile, Panamá e Peru. Houve variação significativa nas taxas entre cidades e subcidades, mesmo depois de considerar o país. Nos modelos totalmente ajustados, as pontuações mais altas de educação na subcidade e o PIB mais alto da cidade foram associados a uma menor taxa de homicídio entre homens e mulheres (Risco Relativo) por cada desvio-padrão maior em homens e mulheres, respectivamente, 0,87 (IC 0,84-0,90) e 0,90 (IC 0,86-0,93) para educação e 0,87 (IC 0,81-0,92) e 0,92 (IC 0,87-0,97) para PIB). O índice de Gini mais alto nas cidades foi associado a taxas de homicídio mais altas (RR 1,28 (IC 1,10-1,48) e 1,21 (IC 1,07-1,36) em homens e mulheres, respectivamente). Maior isolamento também foi associado a maiores taxas de homicídio (RR 1,13 (IC 1,07-1,21) e 1,07 (IC 1,02-1,12) em homens e mulheres, respectivamente).

**Interpretações:** Fatores das cidades e subcidades estão associados às taxas de homicídios. Melhorias na educação, nas condições sociais e na integração física das cidades e na mitigação das desigualdades podem contribuir para a redução dos homicídios na região.

**Financiamento:** the Wellcome Trust [205177/Z/16/Z]

**Keywords:** Homicídios; Saúde Urbana; América Latina; Mortalidade; Forma Urbana; Fatores Sociais
